# Antiviral immunity of severe fever with thrombocytopenia syndrome: current understanding and implications for clinical treatment

**DOI:** 10.3389/fimmu.2024.1348836

**Published:** 2024-04-05

**Authors:** Yuxin Niu, Yunhui Liu, Lanyue Huang, Wei Liu, Qiuyu Cheng, Tingting Liu, Qin Ning, Tao Chen

**Affiliations:** Department of Infectious Diseases, Tongji Hospital, Tongji Medical College and State Key Laboratory for Diagnosis and Treatment of Severe Zoonostic Infectious Disease, Huazhong University of Science and Technology, Wuhan, Hubei, China

**Keywords:** SFTS, antiviral immunity, immune cells, immune response, clinical treatment

## Abstract

Dabie Banda virus (DBV), a tick-borne pathogen, was first identified in China in 2009 and causes profound symptoms including fever, leukopenia, thrombocytopenia and multi-organ dysfunction, which is known as severe fever with thrombocytopenia syndrome (SFTS). In the last decade, global incidence and mortality of SFTS increased significantly, especially in East Asia. Though previous studies provide understandings of clinical and immunological characteristics of SFTS development, comprehensive insight of antiviral immunity response is still lacking. Here, we intensively discuss the antiviral immune response after DBV infection by integrating previous ex- and in-vivo studies, including innate and adaptive immune responses, anti-viral immune responses and long-term immune characters. A comprehensive overview of potential immune targets for clinical trials is provided as well. However, development of novel strategies for improving the prognosis of the disease remains on challenge. The current review may shed light on the establishment of immunological interventions for the critical disease SFTS.

## 1 Introduction

Severe fever with thrombocytopenia syndrome (SFTS) was discovered in China in 2009 (1) as a burgeoning severe hemorrhagic fever disease caused by severe fever with thrombocytopenia syndrome virus (SFTSV) infection, which was formally renamed family *Phenuiviridae*, genus *Bandavirus*, species *Dabie bandavirus* (DBV) in 2019 (2). SFTS has subsequently emerged in Japan, South Korea, and Vietnam. SFTS generally occurs between March and November, with the highest incidence from May through July (3, 4). The tick species *Haemaphysalis longicornis* is a major vector of SFTS and is responsible for exposing over 40% of China’s population in 1140 counties (5, 6). The detection of DBV-specific antibodies and RNA in some animals besides ticks suggests that they may also participate in disease transmission (7–10).

The existing body of research on the clinical manifestations of the disease reveals that patients with SFTS develop a fever with temperature higher than 38°C, thrombocytopenia, lymphadenectasis, gastrointestinal discomfort, and lymphadenopathy. In severe cases, acute multiple organ failure leads to mortality. The disease typically follows a course consisting of several stages, including the incubation period, febrile stage, multiple organ failure, remission, and convalescence (3, 4, 11–14). Respiratory distress, signs of bleeding, neurological symptoms, increased levels of lactate dehydrogenase (LDH), aspartate transaminase/alanine transaminase (AST/ALT) ratio, interleukin 6 (IL-6), neutrophil percentage, C-reactive protein/lymphocyte ratio, activated partial thromboplastin time (APTT), and thrombin time (TT) are correlated with poor prognosis and high mortality in individuals diagnosed with SFTS (13, 15–18). Thus, it can be inferred that SFTS is a multifaceted systemic ailment characterized by hepatic impairment, inflammatory response, and anomalous coagulation (3).

## 2 Trigger of antiviral immunity: DBV invasion of the body

**Figure 1:**
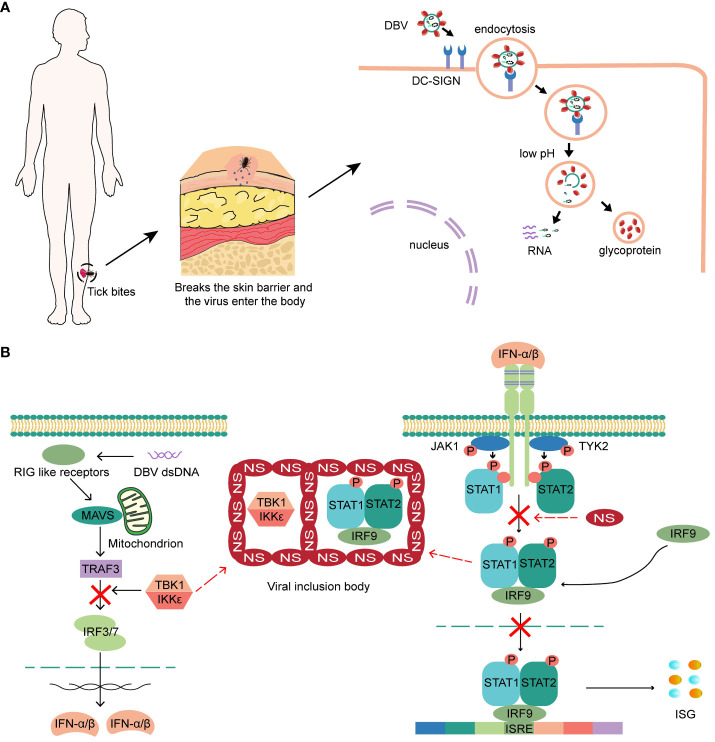
**(A)** The virus attaches to the surface of host cells, and subsequently enters cells. Low pH triggers the fusion of the virus with the cell membrane and releases the viral ribonucleic protein complex into the cytoplasm. **(B)** NSs of DBV can hinder the production and response of intracellular IFN by encapsulating the STAT heterodimer and TBK1/IKKϵ, and block the phosphorylation of STAT2 and nuclear translocation of STAT, ultimately leading to a complete blockade of the IFN response.

Ticks carrying DBV transmit the virus to humans through infestation. The virus attaches to the surface of host cells through interactions between glycoproteins and membrane factors, such as Dendritic cell-specific ICAM-3 grabbing non-integrin (DC-SIGN), and subsequently enters cells through the clathrin-dependent pathway (4, 19–21). (**Figure 1A**). Recently, CCR2 has been discovered as DBV entry binding receptor to virus glycoprotein N (22). Skin-resident cells surrounded the bite location, including keratinocytes, mast cells, immature Langerhans cells and epidermal dendritic cells, are hypothesized to be the first target cell for DBV infection and transmission. Mast cells in skin mucous membranes are found as sentinels for tissue injury, DBV invasion (23) and local responses by degranulating and releasing pre-stored histamine and other bioactive substances within minutes or *de novo* synthesized mediators and lipid mediators within hours (24, 25).

Both live or UV-inactivated DBV virions can stimulate mast cells for releasing bioactive mediators to enhance endothelial and vascular permeability (24). Loss of tight junction between endothelial cells allows a huge amount of white blood cells to exude the site of viral invasion with enhanced immune response. In the early stages of SFTS resulting from tick bites, mast cells play a crucial role in boosting the body’s defense response by producing various bioactive chemicals.

## 3 Innate immunity in DBV infection

The host innate immune response exhibits an initial response to pathogenic microorganisms and acts as a crucial component of the adaptive immune response. Interferon, monocytes, macrophages, and NK cells are identified as critical responders of antiviral innate immunity.

### 3.1 DBV NSs block the interferon response

Interferon performs various roles in innate and acquired immunity, exhibiting potent and universal antiviral activity (26). After infecting epithelial cells, fibroblasts, and pDCs surround the bite location, the immune system responds by secreting interferon-α (IFN-α) and IFN-β. IFN-γ is produced by activated macrophages and NK cells in the early stages or by activated Th1 cells in the later stages, thus inducing an antiviral state. DBV binds to pattern recognition receptors on the cell surface and activates critical kinases that initiate type I interferon (IFN-I) production (27). Secreted IFN then binds to IFNARs on the cell surface, activating JAK-STAT signaling and increasing the expression of antiviral interferon-stimulated genes (ISGs) (28). However, the nonstructural proteins (NSs) of DBV can hinder the production and response of intracellular IFN by encapsulating the STAT heterodimer and TBK1/IKKϵ (29, 30) that are integral molecules of the IFN-I production pathway to form viral inclusion bodies. NSs can also block the phosphorylation of STAT2 and nuclear translocation of STAT, ultimately leading to a complete blockade of the IFN response (4, 31, 32) (**Figure 1B**). Recently discovered a higher prevalence of exacerbated IFN-I signaling pathways and increased ISGs expression in patients with fatal SFTS, and a direct correlation between enhanced ISGs expression and disease severity. Therefore, an increased IFN-I response may be detrimental rather than beneficial (33, 34). The blockade of the interferon response seems to occur only in DBV-infected cells, whereas the majority of cells in SFTS patients still show a high enrichment of the IFN-I signaling pathway, which may contribute to the exacerbation of the disease.

### 3.2 Intermediate phenotype transition of monocytes

Peripheral monocytes are derived from bone marrow and express a variety of receptors that exert strong phagocytosis and pathogen-clearing abilities. They are believed playing pivotal role during expansion of DBV replication via being directly infected by the virus (22, 35, 36). In the early stages of infection, monocytes suffer extensive apoptosis and result in viral particle release (37), which might be contributed by immune surveillance to clear virus-infected target cells. Previous analysis has revealed that intermediate monocytes are more prone to DBV infection than classical monocytes. In vitro, transition of monocytes from the classical to the intermediate phenotype was observed by DBV stimulated peripheral blood mononuclear cells of patients with SFTS, especially in deceased patients (33). The expression of RNA virus infection acquired gene CTSL and CTSB was found significantly increase in intermediate monocytes (33, 38, 39), which may explain why intermediate monocytes are more susceptible to DBV infection. Meanwhile, intermediate monocytes exhibit enhanced expression of ISGs and IFN-dependent chemokines, as well as complement activation. Overactivation of type I IFN response and complement cascade reaction by intermediate monocytes is the hallmark of DBV induced organism injury and indicator of poor prognosis (33). Furthermore, monocytes isolated from individuals with acute SFTS produce significantly lower TNF-α levels by LPS stimulation. This might indicate that monocytes are not major source of TNF-α in SFTS patients (35).

### 3.3 Macrophages M2 phenotype differentiation

Circulating monocytes can migrate to inflammatory tissues for differentiating to macrophages during viral infections, which further activate and release proinflammatory mediators. The function of macrophages could be significantly promoted by IFN-γ with expressing iNOS enzyme and NO to kill virus-infected cells. Based on the specific staining of DBV-positive macrophages of spleen, macrophages are hypothesized to be one of important target cells for DBV infection. Consistently, replication of DBV is observed in primary mouse macrophages in vitro infection model, without affecting phagocytic activity to platelets (40). Another study reported that DBV infection significantly increased the expression of miR-146a and miR-146b in macrophages and facilitated their differentiation into M2 phenotypes (41). M2 macrophages can enhance phagocytic activity but suppress the production of proinflammatory cytokines and reduce pathogen-killing capability (42), which may promote the expansion of DBV (4, 43). In the early stage of DBV infection, elevated expression of IFN-γ encourages macrophages M1 phenotype transition with proinflammatory role via STAT1 pathway, while the elevated DBV replication and IL10 levels promote macrophage M2 differentiation in the late stage of infection (4, 41, 44).

### 3.4 Activation and exhaustion of NK cells

NK cells are one of primary responders of innate immunity to viral infections (4). The surface expression of major histocompatibility complex class I molecules on infected host cells is generally downregulated during viral infection, which can be recognized by NK cells, leading to the direct elimination of infected cells. In addition, NK cells secrete proinflammatory cytokines and aid in the early elimination of the virus. Recent studies have highlighted a negative association between NK cell depletion and severe SFTS, particularly during the early stages of infection (45, 46). CD56^dim^CD16^+^ NK cells are the primary subset of cytotoxic NK cells and their decrease in patients with SFTS may impair the clearance of virus-infected cells and immunomodulation. Furthermore, the activation and functional enhancement of CD56^dim^CD16^+^ NK cells have also been observed in the acute phase of SFTS, with high expression of Ki-67 and GZMB and relatively low expression of NKG2A (47, 48).

### 3.5 Upregulated complement-related gene expression but downregulated protein levels

Activation of the complement system and coagulation disorders can increase the risk of infection caused by various pathogens (49). Complement activation is well known in patients with disseminated intravascular coagulation, and there is a connection between the complement and coagulation systems (50). A recent study examining serum proteins in SFTS patients found a decrease in complement system proteins in those who passed away, except for MASP2. Conversely, complement proteins C4a, C4b, C1s, and C1R were elevated, while C6 and C7 proteins were reduced in recovering patients (51, 52). Therefore, the fatal patients are thought to have deficiencies in their innate immune responses, including the down-regulation of the complement system that leads to the progression of DIC characterized by coagulation dysfunction. Interestingly, another study reported upregulated expression of complement-related genes in patients who died of SFTS (33). This phenomenon may be caused by excessive activation of the complement system and subsequent depletion of complement components. Complement-dependent cytotoxicity undoubtedly assists the body in clearing viruses.

## 4 Adaptive immunity in DBV infection

Patients with SFTS experience decreased lymphocyte blood counts with a significant portion of their lymphocytes are activated and exhibit enhanced functionality, suggesting that these patients mount a robust adaptive immune response against DBV (53). The destruction of antiviral immunity in these patients occurs due to the combined damage inflicted on B and T cells.

### 4.1 B cells activated by DBV act as target cells for viral replication, leading to systemic dissemination

Pathological examination of fatal SFTS cases revealed that large hematopoietic cells in lymphoid organs (lymph nodes, spleen, and bone marrow) comprise the majority of the cells infected by the virus. Additionally, mature lymphocytes are particularly sensitive to DBV infection in fatal cases, with a notable presence of DBV^+^ mononuclear cells found in the capillaries of non-lymphoid organs in deceased patients. The infected cells can be recognized through the detection of plasmablast markers (MUM1 and CD38). However, unlike B cells infected with DBV, DBV^+^ cells in these capillaries do not express CD20 (54). CD20 is a familiar marker for B cells that starts to express in late pre-B lymphocytes (but not in pre-B lymphocytes) and is absent from terminally differentiated plasmablasts and plasma cells (55). Another study showed that most mature B cells infected with DBV in the lymph nodes were activated and had immunophenotypes similar to those of plasmablasts (54, 56). Based on these findings, it can be inferred that B cells at different stages of plasma cell differentiation serve as sites for DBV replication and spread the virus to non-lymphoid organs (54, 57). Elevated proportions of plasmablasts with functional impairments have also been observed (33), resulting in the absence of DBV-specific IgG antibodies against glycoprotein and nucleocapsid protein. Various factors, including impaired DC differentiation and antigen presentation function, failed follicular helper T cell differentiation and B cell antibody class switching, lead to comprehensive impairment of humoral immunity and affect virus clearance (37, 58).

Although DBV infection can activate B cells and cause them to replicate at a low level, most activated B cells are not infected with DBV. Despite limited viral infection and replication efficiency in peripheral blood B cells, the B cell lineage contributes to B cells activation (56). The sirtuin and IFN signaling pathways exhibit broad induction in the whole B cell population of patients who die of the disease, whereas the infected B cell population shows a specific reduction in these pathways compared to the uninfected population (57). This may be due to uninfected immune cells attempting to activate the IFN and sirtuin pathways to against virus. DBV infection of B cells induces uninfected B cells to produce cytokines and chemokines, such as IL-6 *in vitro (* 56). However, DBV-infected B cells can block their signaling pathways and hamper paracrine effects on neighboring uninfected cells *in vivo* ultimately allowing high viral replication (33, 57). This may help DBV evade the host immune system (59).

### 4.2 Decreased T cell counts accompanied by highly active and exhausted phenotypes

DBV infection impairs DCs differentiation and function (58) hindering the development of T follicular helper cells and compromising the effectiveness of virus-specific humoral response (37). T-cell depletion is prevalent during the initial stages of DBV infection, particularly in patients with severe underlying conditions (46). Moreover, changes in T lymphocytes are similar to the overall dynamics of lymphocytes. CD4+ T cell deficiency and Th2 and Th17 bias are strongly correlated with SFTS severity (60). The significant decrease of peripheral T cells is revealed as its apoptosis via the Fas/FasL pathway in patients with SFTS (53). High levels of PD-1 expression are detected in exhausted T cells (61–63) in some chronic viral infections. Approximately 20% of CD4+ and CD8+ T cells display increased PD-1 expression, enhanced cytotoxicity, and effector functions in the early stages of DBV infection (53, 64). Additionally, CD8+ T cells that are similar to the response of antigen-specific T cells during acute viral infections exhibit a stronger response than CD4+ T cells (65). They can be activated rapidly and are recruited mainly by intermediate monocytes via CXCL10 (66). The differentiation from classical to intermediate monocytes in SFTS patients may lead to enhanced CD8+ T cell recruitment and an extensive immunological response. Cellular immunity is critical for virus clearance on antiviral immunity. However, T cell apoptosis, exhaustion, and decreased numbers occur with disease progression, indicating potential impairment of cellular immunity (64).

## 5 DBV replication and clearance and patient prognosis of SFTS

After the virus enters the human body, monocytes, macrophages and mature B cells are promptly infected which lead to extensive viral replication and expansion (22, 54). Subsequently, classical monocytes differentiate into intermediate monocytes with a decrease in CCR2 expression (33, 67). Severe apoptosis of monocytes in the early stages of the infection may result in the release of large amounts of viral particles, leading to the persistent elevation of the viral load in the peripheral blood. At the same time, B cells may experience differentiation blockage under the infection of DBV, and significantly elevated number of plasmablasts with defective antibody secretion function developed which consistently result in humoral immunity insufficient and low level of viral clearance (37). The DBV is eventually cleared from the body through the combined activation of the complement system, NK cells and T cells within 30 days of onset in recovered patients (68). However, patients with advanced age, high viral loads, and concomitant severe underlying diseases and comorbidities are more likely to succumb. Patients recovering from SFTS rarely experience long-term complications, and the levels of DBV-specific IgM antibodies in their bodies decrease over time, becoming undetectable after six months. In contrast, DBV-specific IgG antibodies peaked six months after recovery, then steadily declined but remained detectable for more than eight years. IgG antibodies are found maintaining high levels for a long period and play protective roles against DBV reinfection (68). These findings align with the only one reported case of DBV reinfection. The patient’s second attack was less severe than the first, possibly because the patient had low levels of DBV IgG antibodies compared to other patients treated at the same hospital (69). Individuals who recover from DBV infection typically generate protective IgG antibodies that are unique to the virus. However, low levels of these antibodies diminish their protective effects and lead to reinfection in rare cases.

## 6 Discussion

**Figure 2:**
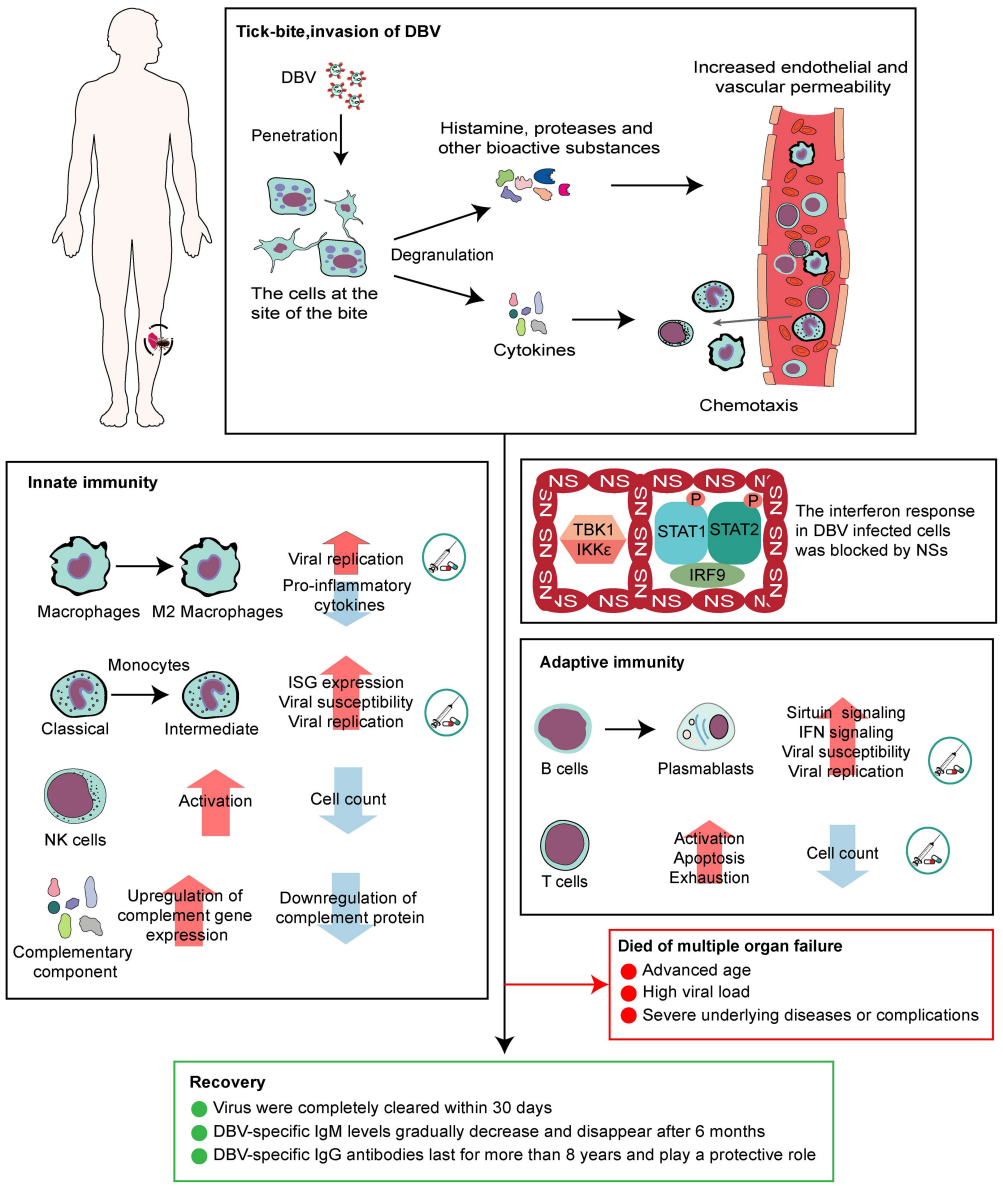
Antiviral immunity and patient prognosis of SFTS.

A complete description of the immune response in DBV-infected people is required to identify possible targets for therapeutic intervention. In conclusion, cells at the site of infection begin antiviral immunity. DBV can influence macrophage activation and differentiation (41), activate NK cells but reduce their numbers (47), and alter the monocyte phenotype into intermediate monocytes. B cells might be prevented from differentiating into plasma cells, increasing the frequency of plasmablasts with IFN-I response-related pathways. The function of CD4+ and CD8+ T cells is enhanced in SFTS patients accompanied with apoptosis via the Fas/FasL pathway (53) (**Figure 2**). Based on this, we proposed some potential intervention strategies, including lowering the susceptibility of monocytes, macrophages, and mature B cells to DBV, inhibiting viral replication within these cells, and improving T cell apoptosis and depletion to facilitate the body’s virus clearance, which requires a clear understanding of the specific mechanisms by which DBV enters different cells. Currently, it is accepted that DBV can interact with CCR2 to enter monocytes, and *in vitro* experiments have shown that CCR2 inhibitors can inhibit viral replication (22). The mechanisms by which the virus infects macrophages and B cells and T cell exhaustion remain unknown, making treatment intervention for SFTS a significant challenge.

Available clinical trials of SFTS have mainly focused on broad-spectrum antivirals (**Supplementary Material**). Favipiravir and ribavirin may be more effective in patients with low viral loads (70, 71), suggesting the need for the early administration of antiviral agents. In addition, favipiravir has not shown any benefit in patients older than 70 years (70). Retrospective studies have demonstrated that IFN-α therapy has no significant therapeutic effect on SFTS (33). Although arginine therapy may boost T cell activity by stimulating the restoration of CD3-ζ chain expression, its clinical application merely accelerates platelet and AST/ALT normalization without enhancing patient survival rates (72). Most clinical trials have shown limited efficacy in treating SFTS patients, underscoring the importance of developing targeted therapies for SFTS patients and the need for further investigation into potential treatment options. It is crucial to continue advancing our understanding of SFTS and to develop effective clinical interventions to improve patient outcomes.

## Author contributions

YN: Writing – original draft. YL: Investigation, Writing – review & editing. LH: Investigation, Writing – review & editing. WL: Investigation, Writing – review & editing. QC: Investigation, Writing – review & editing. TL: Investigation, Writing – review & editing. QN: Writing – review & editing. TC: Writing – review & editing.
